# NPP-21/TPR is required for developmental control of spindle checkpoint strength in *C. elegans*

**DOI:** 10.64898/2026.04.13.718277

**Published:** 2026-04-15

**Authors:** Natalie Gallagher, Shevelle Brown, Valentin Duprat, Simone Köhler, Abby F. Dernburg, Needhi Bhalla

**Affiliations:** # Department of Molecular, Cell and Developmental Biology, University of California, Santa Cruz, Santa Cruz, CA 95064; * Department of Molecular and Cell Biology, University of California, Berkeley, Berkeley, CA 94720; § Current address: Cell Biology and Biophysics Unit, European Molecular Biology Laboratory, 69117 Heidelberg, Germany

## Abstract

The spindle checkpoint ensures accurate chromosome segregation by monitoring whether chromosomes, via kinetochores, are properly attached to the mitotic or meiotic spindle. If chromosomes are unattached, the checkpoint delays the cell cycle to facilitate error correction. In *C. elegans* early embryos, activation of the spindle checkpoint produces a longer mitotic delay in cells that will become germline tissue than in cells that will become somatic tissue, suggesting developmental regulation. We show that the conserved nucleoporin and spindle matrix component, NPP-21/TPR, is required for the stronger spindle checkpoint in germline cells. A strain expressing AID-3xFLAG-NPP-21 and the Arabidopsis TIR1 gene cannot support the stronger checkpoint in germline cells. When AID-3xFLAG-NPP-21 is acutely degraded, the checkpoint in germline cells is further compromised while somatic cells are unaffected. A checkpoint-proficient NPP-21-GFP transgene localizes to a spindle-like structure during mitosis and is enriched in germline cells, consistent with a cell-fate specific function for this protein. Finally, NPP-21 controls spindle checkpoint strength in germline cells by two, potentially linked, control points: concentrating PCH-2 around mitotic chromosomes and promoting the localization of the checkpoint effector, Mad2, to unattached kinetochores. These experiments demonstrate a developmental role for NPP-21, and the spindle matrix, in controlling spindle checkpoint strength in immortal germline cells in *C. elegans*.

## Introduction

The spindle checkpoint is a cell cycle surveillance mechanism that ensures that chromosomes are attached to the mitotic or meiotic spindle to guarantee accurate chromosome segregation and prevent aneuploidy. When chromosomes are unattached, a soluble, molecular signal halts or delays the cell cycle to allow for the defect to be corrected. Defects in spindle checkpoint components are associated with cancer progression (Kops et al., 2005; Matthews et al., 2022) and infertility (Baker et al., 2004; Huang et al., 2023; Zhang et al., 2020), highlighting their importance to human health.

The spindle checkpoint initiates from unattached kinetochores, the specialized structures that assemble on chromosomes’ centromeres to mediate microtubule attachment and proper segregation (Lara-Gonzalez et al., 2021). A complex of the conserved checkpoint factors, Mad1 and Mad2, binds unattached kinetochores. Mad2 adopts two functionally distinct conformations: an open, inactive form and a closed, active form. Mad2’s conversion from inactive to active occurs when Mad2 binds a specific motif, called the closure motif, in binding partners. Mad2 binding partners, such as Mad1 and Cdc20, each have closure motifs and thus, catalyze the conversion of Mad2 from inactive to active. Once bound to unattached kinetochores, the Mad1/Mad2 complex recruits additional inactive Mad2 so that it can bind Cdc20, adopting the active conformation and forming the Mitotic Checkpoint Complex, or MCC. The MCC sequesters Cdc20, a factor required to promote anaphase, to inhibit the cell cycle and allow time for error correction (Lara-Gonzalez et al., 2021). The evolutionarily ancient ATP-ase PCH-2/TRIP13 plays a conserved role promoting spindle checkpoint function by ensuring the availability of open, inactive Mad2 (Ma and Poon, 2016; Ma and Poon, 2018; Nelson et al., 2015)

Despite the important role of the spindle checkpoint in preventing aneuploidy, the mitotic delay or arrest installed by the spindle checkpoint can be highly variable. The variability of the cell cycle delay is called spindle checkpoint strength and depends on the number of unattached kinetochores, cell volume, and cell fate (Collin et al., 2013; Galli and Morgan, 2016; Gerhold et al., 2018; Kyogoku and Kitajima, 2017). For example, during early embryogenesis in *C. elegans*, cells that are destined to become germline tissue have a stronger checkpoint than their somatic counterparts, independent of their smaller size (Galli and Morgan, 2016; Gerhold et al., 2018), demonstrating developmental differences in checkpoint strength. We have shown that the stronger checkpoint in germline cells depends on the asymmetric enrichment of the spindle checkpoint factor, PCH-2/TRIP13 (Defachelles et al., 2020), likely through PCH-2/TRIP13’s ability to ensure more open, inactive Mad2 for increased MCC production.

We wanted to identify additional factors that might also contribute to the stronger checkpoint in germline cells of the early embryo. We noticed that after nuclear envelope breakdown, PCH-2-GFP and other checkpoint components, such as GFP-Mad1 and GFP-Mad2, formed a “cloud” around mitotic chromosomes (Defachelles et al., 2020; Essex et al., 2009). This “cloud” sometimes resembled a controversial mitotic structure called the spindle matrix (Zheng, 2010), especially in germline cells. The spindle matrix has been observed in both *Drosophila* and human somatic cells, but both its composition and essential function have been unclear. Some spindle matrix components interact with spindle checkpoint components (Lince-Faria et al., 2009; Schweizer et al., 2013), making them attractive candidates to potentially regulate spindle checkpoint strength during development.

NPP-21/TPR is the *C. elegans* ortholog of the *Drosophila* spindle matrix protein, Megator, and the human TPR. In *C. elegans*, NPP-21/TPR has been primarily characterized as a nucleoporin, a member of a family of proteins which assemble the nuclear pore and allow for molecular transport between the cytoplasm and nucleus (Cohen-Fix and Askjaer, 2017). NPP-21/TPR forms the nucleoplasmic face or basket of the pore (Cohen-Fix and Askjaer, 2017). In *Drosophila*, Megator is a nucleoporin but also localizes to the area occupied by the miotic spindle (Qi et al., 2004) and supports activation of the spindle checkpoint by ensuring robust recruitment of Mad2 to unattached kinetochores (Lince-Faria et al., 2009). We hypothesized that NPP-21 may play a similar role during development in *C. elegans* and that this role might be particularly relevant in germline cells during early embryogenesis.

Here, we show that NPP-21/TPR, in its capacity as a spindle matrix component, is required for developmental control of spindle checkpoint strength in *C. elegans*. A strain carrying both a transgene of *npp-21*, *AID-3xFLAG::npp-21*, and the Arabidopsis TIR1 gene is functional for checkpoint activation in somatic cells of the early embryo but cannot support the stronger checkpoint in germline cells, suggesting that the stronger checkpoint in these cells is sensitive to subtle changes in NPP-21 protein levels. When the tagged protein is acutely degraded, checkpoint strength is further compromised in germline cells but has no effect on somatic cells. Consistent with this cell-fate specific function, NPP-21-GFP consistently forms a structure that resembles the spindle matrix during mitosis and is asymmetrically enriched in germline cells, compared to somatic cells. Finally, NPP-21 ensures a stronger checkpoint in P_1_ cells by concentrating PCH-2 around mitotic chromosomes and promoting the robust recruitment of Mad2 to unattached kinetochores.

## Results and Discussion

### NPP-21 is required for the stronger spindle checkpoint in germline, P_1_ cells

NPP-21 is an essential gene. Therefore, to determine whether NPP-21 plays a role in the spindle checkpoint in early developing embryos, we exploited the auxin-inducible degron system (AID) to acutely degrade NPP-21 in oocytes and early embryos. We generated a version of NPP-21 tagged at its N-terminus with the AID and a 3xFLAG tag (*AID-3xFLAG::npp-21*, also referred to as *AID::npp-21*) in a strain that expresses the Arabidopsis TIR1 gene under a germline promoter (*Pgld-1::TIR1-mRuby)* (Zhang et al., 2015). TIR1 is necessary to induce rapid degradation of tagged proteins upon exposure to auxin. This tagged version of NPP-21, at least in the presence of the TIR1 gene, mildly affects embryonic viability and meiotic chromosome segregation, as *AID::npp-21* worms grown on ethanol produce inviable embryos and males at a higher frequency than control worms exposed to either ethanol or auxin (Table 1). Since hermaphrodites are XX and males are XO, an increase in the number of male self-progeny is indicative of defects in meiotic chromosome segregation. When *AID::npp-21* worms were exposed to auxin, very few viable progeny were produced and among the viable progeny, there was a significant increase in male self-progeny (Table 1). Thus, the *AID::npp-21* transgene is mostly functional, with some slight defects in embryonic viability and meiotic chromosome segregation, which may be the product of the tag and/or inappropriate TIR1 activity. By contrast, auxin-induced depletion of *AID::npp-21* results in very high embryonic lethality and severe defects in meiotic chromosome segregation, consistent with its essential role.

**Figure 1:**
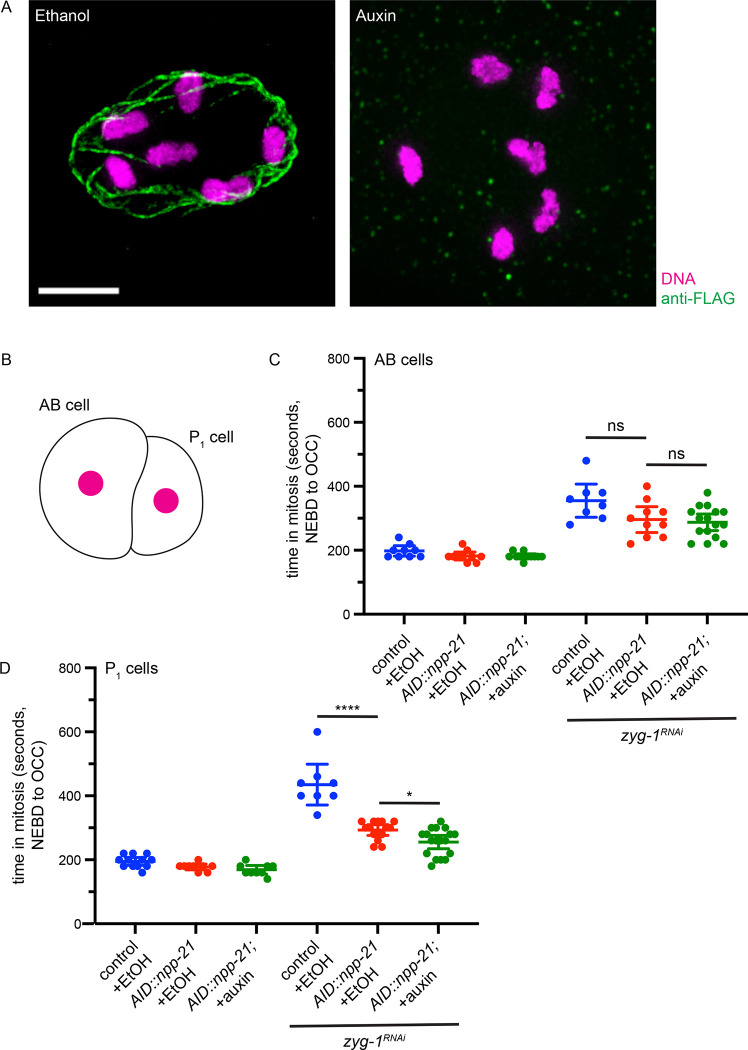
NPP-21 is required for the stronger spindle checkpoint in germline cells of the two-cell embryo. A. AID-3xFLAG-NPP-21 is degraded in oocytes after one hour of auxin treatment. Images of cellularized (−1) oocytes from worms treated with ethanol or auxin stained with antibodies against the FLAG tag and DAPI to visualize DNA. Scale bar indicates 5 microns. B. Cartoon indicating a two cell embryo with the AB and P_1_ cell labeled and the nucleus colored magenta. C. AID-3XFLAG-NPP-21 is functional and not required for the spindle checkpoint in AB, somatic, cells. Graph depicting mitotic timing in AB cells of embryos treated with ethanol or auxin and control RNAi or *zyg-1* RNAi. D. AID-3XFLAG-NPP-21 cannot support the stronger checkpoint and is required for a robust spindle checkpoint in P_1_, germline, cells. Graph depicting mitotic timing in P_1_ cells of embryos treated with ethanol or auxin and control RNAi or *zyg-1* RNAi. A **** indicates a p value < 0.0001, a * indicates a p value < 0.05 and all error bars indicate 95% confidence intervals.

We tested whether we could reliably and rapidly degrade AID-3xFLAG-NPP-21. We performed this experiment in the most proximal oocytes undergoing cellularization (−1 oocyte). Upon fertilization, these oocytes complete meiosis and enter mitosis to begin embryonic development within an hour (Robertson and Lin, 2013). We exposed young adult hermaphrodites worms undergoing oogenesis to auxin for one hour and performed indirect immunofluorescence against the FLAG tag to visualize NPP-21. In worms exposed to ethanol, we observed staining surrounding the chromosomes in the most proximal oocytes, consistent with the protein being a member of the nuclear pore complex in the nuclear envelope (Figure 1A). After one hour of treatment, we could no longer detect AID-3xFLAG-NPP-21 at the nuclear periphery (Figure 1A), indicating that we can reliably and rapidly degrade NPP-21 in oocytes that are about to be fertilized and develop into embryos.

Having established this protocol, we used it to test whether degradation of NPP-21 affected normal mitotic timing and spindle checkpoint function in early embryos, specifically two cell embryos which have already initiated the process of cell differentiation into soma (AB cells) and germline (P_1_ cells) tissue (Figure 1B). We analyze mitotic timing by calculating the time between nuclear envelope breakdown (NEBD) and the onset of cortical contractility (OCC) in embryos that have an mCherry tagged histone, mCherry-H2B, and the plasma membrane marker, GFP-PH (Essex et al., 2009). NEBD is visualized as the diffusion of soluble mCherry-H2B signal from the nucleus and OCC is detected when the plasma membrane changes conformation from circular to rectangular. We performed this experiment in both AB and P_1_ cells since these two different cells vary in both cell fate and spindle checkpoint strength (Figure 1B). P_1_ cells have a stronger checkpoint than their somatic counterparts and this is dependent on conserved factors required for its developmental fate, independent of its smaller size (Galli and Morgan, 2016; Gerhold et al., 2018). When transgenic *AID::npp-21* worms were exposed to ethanol or auxin, we did not detect any difference in mitotic timing between ethanol-treated or auxin-treated worms, or with control worms treated with ethanol, in either AB or P_1_ cells, indicating that NPP-21 is not required for unperturbed mitosis in either cell type (Figures 1C and D).

Next, we activated the spindle checkpoint in *AID::npp-21* AB cells by performing RNA interference (RNAi) against *zyg-1*, a conserved cell cycle kinase required for centrosome duplication (O’Connell et al., 2001). In the absence of *zyg-1*, two cell embryos only have one centrosome and generate a monopolar spindle, producing unattached kinetochores and spindle checkpoint activation (Essex et al., 2009). In AB cells of *zyg-1*^*RNAi*^ embryos, we observed a slight decrease in mitotic timing in transgenic *AID::npp-21* worms on ethanol or auxin (Figure 1C), when compared to control embryos on ethanol. This difference was not statistically significant, indicating that NPP-21 might play a role in promoting a strong spindle checkpoint in AB cells but is not required for the checkpoint in these cells. In contrast, when we activated the spindle checkpoint in P_1_ cells in *AID::npp-21* worms exposed to ethanol, these cells showed similar mitotic timing as AB cells treated with *zyg-1* RNAi (Figures 1C and D) and a weaker checkpoint than control worms (Figure 1D). These data indicate that either the transgenic *AID::npp-21* is not fully functional for spindle checkpoint function in P_1_ cells or that the presence of TIR1, even in the absence of auxin, affects protein function. More importantly, these data suggest a role for NPP-21 in spindle checkpoint strength in this specific cell type. When *AID::npp-21* worms are exposed to auxin for more than one hour, we detected a further weakening of the spindle checkpoint (Figure 1D), indicating that NPP-21 is required for a robust spindle checkpoint specifically in P_1_ cells.

### NPP-21-GFP adopts a structure similar to the spindle matrix and is enriched in P_1_ cells

**Figure 2:**
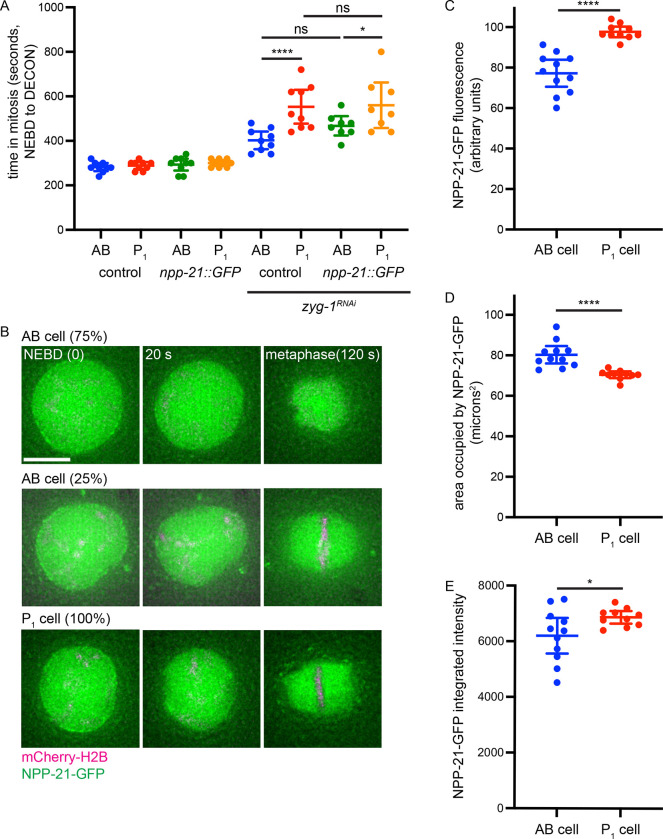
NPP-21-GFP is enriched in P_1_, germline, cells and its localization is distinct from that in AB, somatic, cells. A. NPP-21-GFP is functional for the spindle checkpoint in AB and P_1_ cells. A graph depicting mitotic timing in AB and P_1_ cells of control and *npp-21::GFP* animals treated with control and *zyg-1* RNAi. B. NPP-21-GFP localization in P_1_ cells is different than in AB cells in most two cell embryos. Images of AB and P_1_ cells expressing mCherry-H2B (magenta) and NPP-21-GFP (green) at NEBD, 20 seconds after NEBD and at metaphase (120 seconds after NEBD). C. GFP-NPP-21 is asymmetrically enriched in P_1_ cells. Graph depicting average fluorescence of NPP-21-GFP in AB and P_1_ cells 20 seconds after NEBD. D. The area occupied by GFP-NPP-21 is smaller in P_1_ cells. Graph depicting the area in microns^2^ of NPP-21-GFP in AB and P_1_ cells 20 seconds after NEBD. E. GFP-NPP-21 is asymmetrically enriched in P_1_ cells. Graph depicting integrated intensity of NPP-21-GFP in AB and P_1_ cells 20 seconds after NEBD. A **** indicates a p value < 0.0001, a *** indicates a p value < 0.001, a * indicates a p value < 0.05 and all error bars indicate 95% confidence intervals.

Given this cell fate-specific difference in the requirement for NPP-21 function in the spindle checkpoint, we localized NPP-21-GFP in AB and P_1_ cells in two cell embryos (Thomas et al., 2023). First, we verified that *npp-21::gfp* worms were competent for checkpoint activation in AB and P_1_ cells. Because the presence of NPP-21-GFP prevented us from using GFP-PH to monitor OCC, we measured mitotic timing using mCherry-H2B from NEBD to decondensation of mitotic chromosomes (DECON) (Essex et al., 2009). Both AB and P_1_ cells in embryos expressing NPP-21-GFP activated the spindle checkpoint and P_1_ cells displayed a stronger checkpoint than AB cells, similar to control worms (Figure 2A), verifying that NPP-21-GFP is fully functional for checkpoint activation. Next, we localized NPP-21-GFP and observed a striking difference in its localization in AB and P_1_ cells. In 75% of two-cell embryos (6/8), NPP-21-GFP remained in a cloud around mitotic chromosomes 120 seconds after NEBD, a timepoint that corresponds to metaphase, in AB cells. In contrast to AB cells, in P_1_ cells, after NEBD, NPP-21-GFP reorganized around mitotic chromosomes in a metaphase plate to adopt a structure that resembled the mitotic spindle, similar to a spindle matrix. In 25% of two cell embryos, both AB and P_1_ cells exhibited this spindle-like localization (Figure 2B). Thus, in P_1_ cells 100% of the time, and in AB cells 25% of the time, NPP-21-GFP adopts a localization pattern during mitosis that resembles the spindle matrix.

We have previously shown that some factors required for spindle checkpoint strength, such as PCH-2-GFP, are enriched in P_1_ cells (Defachelles et al., 2020). To determine whether NPP-21-GFP is also enriched in P_1_ cells, we quantified the average fluorescence of NPP-21-GFP that remained around mitotic chromosomes in the timepoint immediately after NEBD (images labeled 20 seconds in Figure 2B). We performed this quantification at this stage of the cell cycle since this localization was similar in both AB and P_1_ cells immediately after NEBD and entry into mitosis, in contrast to the changes in localization we observed at metaphase in AB and P_1_ cells (Figure 2B). When we performed this analysis, we detected more NPP-21-GFP in P_1_ cells than AB cells (Figure 2C). We were concerned that, even at this early timepoint, the area occupied by NPP-21-GFP was also different in AB and P_1_ cells, potentially affecting this quantification. To test this, we determined the area occupied by NPP-21-GFP signal in AB and P_1_ cells and found that this area was significantly smaller in P_1_ cells than AB cells (Figure 2D). Since AB and P1 cells show similar nuclear areas prior to NEBD (Gerhold et al., 2018), this indicates that NPP-21-GFP is regulated differently in P_1_ cells even at this early timepoint. However, when we calculated the integrated intensity of NPP-21-GFP signal (Figure 2E) in both cell types, NPP-21-GFP signal was still significantly higher in P_1_ cells than AB cells, indicating that NPP-21-GFP is enriched in P_1_ cells, potentially to drive the stronger checkpoint.

### NPP-21 concentrates PCH-2-GFP around mitotic chromosomes

We showed that the stronger checkpoint in P_1_ cells is controlled by the asymmetric enrichment of the conserved ATP-ase, PCH-2, in P_1_ cells (Defachelles et al., 2020). PCH-2 and its mammalian ortholog, Trip13, remodels active Mad2 to generate inactive Mad2 (Ye et al., 2015). This remodeling guarantees the availability of inactive Mad2 during checkpoint activation so that the conversion of inactive Mad2 to active Mad2 is the rate limiting event in spindle checkpoint activation (Ma and Poon, 2016; Ma and Poon, 2018; Nelson et al., 2015). Based on this activity, we proposed that an increase in PCH-2 promotes the stronger checkpoint in P_1_ cells by increasing the amount of available, inactive Mad2 that could contribute to soluble MCC formation and drive a stronger checkpoint response (Defachelles et al., 2020).

**Figure 3:**
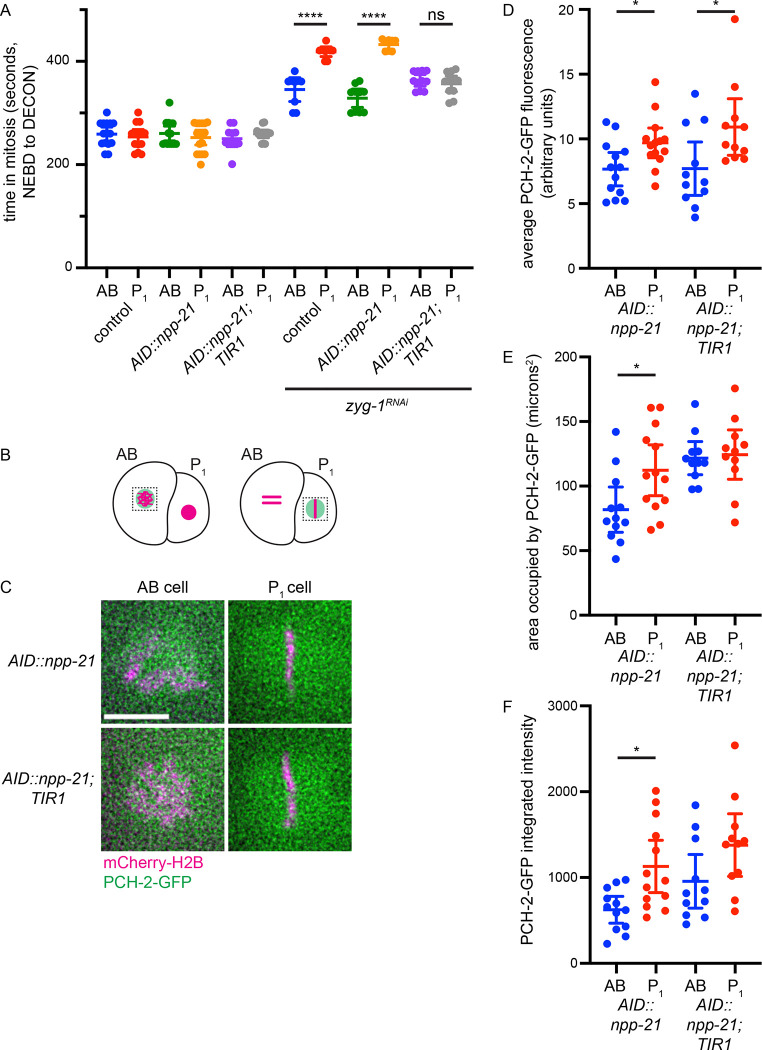
NPP-21 concentrates PCH-2-GFP around mitotic chromosomes in both AB and P_1_ cells. A. Worms expressing both AID-3XFLAG-NPP-21 and TIR1 cannot support the stronger checkpoint. A graph depicting mitotic timing in AB and P_1_ cells of control, *AID::npp-21* and *AID::npp-21;TIR1* animals treated with control and *zyg-1* RNAi. B. Cartoon of PCH-2-GFP localization in AB and P_1_ cells. C. Images of PCH-2-GFP (green) localization in AB and P_1_ cells expressing mCherry-H2B (magenta) in metaphase, in embryos without and with the TIR1 gene. Scale bar indicates 5 microns. D. PCH-2-GFP is asymmetrically enriched in P_1_ cells, in embryos without and with the TIR1 gene. Graph depicting average fluorescence of PCH-2-GFP in AB and P_1_ cells at metaphase. E. The area occupied by PCH-2-GFP is greater in AB and P_1_ cells in embryos expressing TIR1. Graph depicting the area in microns^2^ of PCH-2-GFP in AB and P_1_ cells in metaphase. E. PCH-2-GFP is less concentrated in P_1_ cells of embryos expressing TIR1. Graph depicting integrated intensity of PCH-2-GFP in AB and P_1_ cells. A * indicates a p value < 0.05 and all error bars indicate 95% confidence intervals.

To evaluate whether NPP-21 contributed to the stronger spindle checkpoint in P_1_ cells by regulating PCH-2, we generated a strain that had both *AID::npp-21* and a tagged, functional version of *pch-2*, *pch-2::GFP-3xFLAG* (Nelson et al., 2015). Both *npp-21* and *pch-2* are closely linked (0.20 cM apart). To generate the *AID::npp-21 pch-2::GFP-3xFLAG* double mutant strain, the TIR1 transgene, also on the same chromosome, was crossed off in the recombination event. Since we observed the defect in checkpoint strength in P_1_ cells even in embryos that have not been treated with auxin (Figure 1D), we first determined whether we could perform this experiment in worms that did not express the TIR1 transgene. These double mutants do not exhibit any embryonic inviability, indicating that the embryonic inviability we observe in *AID-3xFLAG::npp-21 Pgld-1::TIR1-mRuby* strains on ethanol was likely the product of inappropriate TIR1 activity (Table 1). *AID-3xFLAG::npp-21 pch-2::GFP-3xFLAG* hermaphrodites did produce males at a higher, statistically significant frequency than *pch-2::GFP-3xFLAG* animals, suggesting a very weak defect in meiotic chromosome segregation (Table 1). However, *AID-3xFLAG::npp-21 pch-2::GFP-3xFLAG* embryos display a stronger checkpoint in P_1_ cells, similar to control animals (Figure 3A), indicating that *AID-3xFlag::npp-21* is fully functional and the weaker checkpoint we observed in *3xFLAG::npp-21 Pgld-1::TIR1-mRuby* P1 cells was a product of the presence of the TIR1 gene.

We introduced *Psun-1::TIR1-mRuby* into strains with *AID-3xFLAG::npp-21 pch-2::GFP-3xFLAG*. Strains carrying AID-NPP-21, this transgenic version of TIR1 and PCH-2-GFP showed a weaker spindle checkpoint in P_1_ cells, similar to AB cells and in contrast to control animals and strains with only AID-NPP-21 and PCH-2-GFP (Figure 3A). This phenotype was also similar to the original strain expressing only AID-NPP-21 and TIR1 (Figure 1D). When these worms were grown on plates containing ethanol, we observed an increase in male self-progeny and no effect on viability (Table 1).

We quantified PCH-2-GFP in both AB and P_1_ cells in embryos expressing only AID-NPP-21 and PCH-2-GFP (no TIR1) or expressing AID-NPP-21, TIR1 and PCH-2-GFP (Figures 3B and C). Both strains with and without TIR1 showed statistically significant enrichment of PCH-2-GFP in P_1_ cells, compared to AB cells (Figure 3D). However, we noticed that the area occupied by PCH-2-GFP increased in both AB and P_1_ cells in strains expressing TIR1 (Figure 3E and Supplemental Figure 1). Moreover, in P_1_ cells without TIR1, the area occupied by PCH-2-GFP was larger than that observed in AB cells without TIR1, likely because PCH-2-GFP often adopted a matrix-like structure in P_1_ cells (55%, 6 of 11 embryos) (Figure 3C and Supplemental Figure 1). However, this structure was much less defined than the one observed with NPP-21-GFP (Figure 2B). When the area occupied by PCH-2-GFP was used to calculate the integrated density of PCH-2-GFP in AID-NPP-21 embryos with and without TIR1, P_1_ cells in strains with TIR1 were still enriched for PCH-2-GFP but the comparison with AB cells was not statistically significant (Figure 3F). This was in direct contrast to the experiments performed in embryos with no TIR1 (Figure 3F). These data indicate that NPP-21 is not required to enrich PCH-2-GFP in P_1_ cells but instead, contributes to its concentration around mitotic chromosomes in both AB and P_1_ cells. Given the defect in checkpoint strength in P_1_ cells, we would argue that this concentration is particularly important for the stronger checkpoint in P_1_ cells. The inability to properly concentrate PCH-2-GFP in P_1_ cells may also explain why PCH-2-GFP rarely adopts a matrix-like structure in P_1_ cells in embryos expressing AID-NPP-21 and TIR1 (18%, 2 of 11 embryos) (Figure 3C and Supplemental Figure 1).

### NPP-21 promotes GFP-MAD-2 recruitment to unattached kinetochores in P_1_ cells

**Figure 4:**
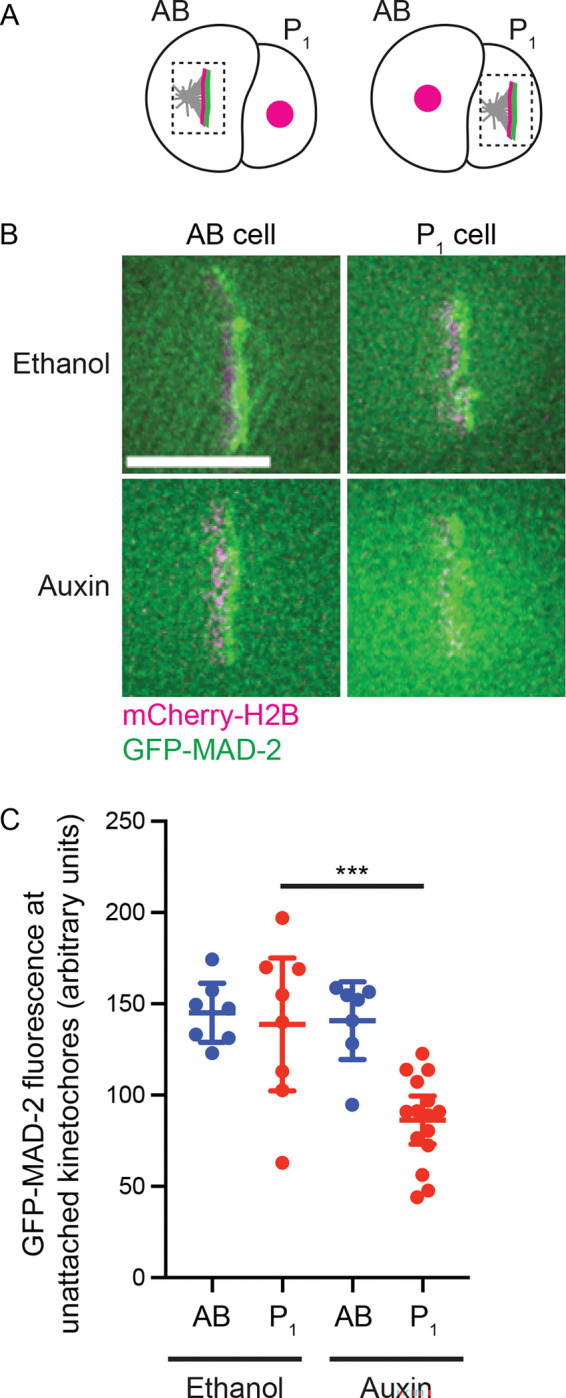
NPP-21 is required for GFP-MAD-2 recruitment to unattached kinetochores in P_1_, germline, cells. A. Cartoon of GFP-MAD-2 localization in AB and P_1_ cells in *zyg-1*^*RNAi*^ embryos. B. Images of GFP-MAD-2 (green) localization in AB and P_1_ cells in *zyg-1*^*RNAi*^ embryos expressing mCherry-H2B (magenta), treated with ethanol (top) or auxin (bottom). Scale bar indicates 5 microns. C. Quantification of GFP-MAD-2 in AB and P_1_ cells at unattached kinetochores in *zyg-1*^*RNAi*^ embryos treated with ethanol or auxin. A *** indicates a p value < 0.001 and all error bars indicate 95% confidence intervals.

In both *Drosophila* and in vitro cell cultured human (HeLa) cells, the NPP-21 ortholog, Megator (*Drosophila*) and TPR (humans), contributes to Mad2, but not Mad1, localization to unattached kinetochores (Lince-Faria et al., 2009; Schweizer et al., 2013). In *Drosophila*, Megator has been proposed to act as a spatial regulator of Mad2 (Lince-Faria et al., 2009) while in human cells, TPR appears to ensure the stability of checkpoint proteins for checkpoint function (Schweizer et al., 2013). To test what effect NPP-21 had on Mad2 recruitment to unattached kinetochores (Figures 4A and B), we quantified GFP-MAD-2 at unattached kinetochores in both AB and P_1_ cells in *AID::npp-21* embryos, treated with either ethanol or auxin (Figures 4B and C). AB cells in embryos treated with ethanol or auxin did not exhibit any difference in GFP-Mad2 recruitment to unattached kinetochores (Figures 4B and C), consistent with the checkpoint activation we observed in these cells (Figure 1C). GFP-Mad2 localization in P_1_ cells treated with ethanol resembled the AB cells we analyzed, indicating that the weaker spindle checkpoint in these cells is not the result of a defect in Mad2 recruitment (Figures 4B and C). By contrast, in P_1_ cells treated with auxin, GFP-Mad2 localization to unattached kinetochores was sharply reduced (Figures 4B and C), demonstrating that the weaker checkpoint response in these cells was explained by the inability to robustly localize Mad2 to unattached kinetochores, similar to what has been reported in *Drosophila* and HeLa cells.

During early development, embryos balance the rapid cell divisions of early embryogenesis with the imperative of maintaining genomic integrity, particularly in immortal germline tissue that will give rise to sperm and eggs. NPP-21, through its spindle matrix function, contributes to this balance by promoting a stronger spindle checkpoint response in germline cells, in contrast to somatic cells. The NPP-21 protein displays germline specific behavior: it is enriched and consistently forms a spindle matrix in P_1_ cells, suggesting a conserved developmental role during embryogenesis. This function acts at two control points: concentrating PCH-2 around mitotic chromosomes and promoting Mad2 recruitment to unattached kinetochores. Given PCH-2’s role in promoting the availability of inactive, open Mad2 for checkpoint signaling, it’s formally possible that these two control points are mechanistically linked. Whether other, conserved spindle matrix components play similar, developmental, roles during embryogenesis is an interesting, open question for our future studies.

## Materials and Methods

### *C. elegans* Strains and Husbandry

The wildtype *C. elegans* strain background was Bristol N2 (Brenner, 1974). All strains were maintained at 20°C. See Supplemental Table 1 for a list of all *C. elegans* strains used in this study.

The AID::3xFLAG::NPP-21 transgenic strain was generated by CRISPR/Cas9 genomic editing (Paix et al., 2017). Young adult worms carrying the *Pgld-1::Tir1mRuby::3’UTR(gld-1) (ieSi64)* transgene were injected with preassembled Cas9-RNP, 23 ng/μL of ssDNA repair template and 5 ng/uL pCFJ90 and 2.5 ng/uL pCFJ104 as co-injection markers. The target crRNA was TAGTCTTTCAGTTATGGATG (IDT) and the ssDNA repair template was generated by primer extension PCR using the following primer GTGTTATCTAATTAAATCTCTCAGTTCCTTCAAGTTATCATAT and gBlock CTCTCAGTTCCTTCAAGTTATCATATTTTTTAAGTAAAACTAATCGCCCGAAATTTAGTCTTTCAGTTATGCCTAAAGATCCAGCCAAACCTCCGGCCAAGGCACAAGTTGTGGGATGGCCACCGGTGAGATCATACCGGAAGAACGTGATGGTTTCCTGCCAAAAATCAAGCGGTGGCCCGGAGGCGGCGGCGTTCGTGAAGGACTATAAAGATCACGACGGAGATTACAAGGACCATGATATCGACTACAAGGACGACGACGACAAGGGAGATGTGGATGCGCCACTTCAGGCGCCTGAACAACCGGTCGCAGACGCTGACGATGAAGAAGCTAACTGGGAAATGGAAAAAGCCGAAATGAAACGGATTGAG (both ordered from IDT). Transformants were identified and worms carrying the transgene were genotyped using the following primers: AATTGGCATCGCTGATCAATGAG and GCTCTTTAATTTCAGCCGCATGAC. The presence of the transgene was verified by sequencing and the transgenic strain was backcrossed at least three time before analysis.

### Viability and fertility

To score total progeny and male self-progeny, L4 hermaphrodites were picked onto individual plates with auxin, ethanol or no additional treatment, and transferred to new plates daily over 4 days. The eggs laid on each plate were counted after removing the parent. Viable progeny and male progeny were quantified when the F1 reached L4 or adult stages (2–3 days post egg laying).

### Microscopy & Mitotic Timing Experiments

All immunofluorescence and live microscopy was performed on a DeltaVision Personal DV deconvolution microscope (Applied Precision) equipped with a 100X N.A. 1.40 oil-immersion objective (Olympus) coupled with a CoolSNAP charge-coupled camera (Roper Scientific). For live microscopy of two cell embryos, eggs were dissected 18–26 hours post-L4 into 1X Egg Buffer (25mM Hepes pH7.4, 118uM NaCl, 48mM KCl, 2mM EDTA, 0.5mM EGTA) and mounted on 2% agarose pads for immediate analysis. Environmental temperature averaged 21°C during image collection for all experiments. For mitotic timing experiments, Z-sections were acquired with 8× 2μm steps using a 100X objective (Olympus) at 20s intervals. Exposure time was 100ms for mCherry-H2B and 50ms for GFP-PH. Mitotic duration was calculated for the AB cell in the presence of monopolar spindles as the interval between NEBD to the onset of cortical contractility (OCC) or the interval between NEBD and chromosome decondensation (DECON). NEBD was defined by the equilibration of mCherry-H2B from the nucleus into the cytosol. OCC was defined as the change in conformation of the plasma membrane from circular to rectangular, or with *zyg-1* RNAi as the first frame when a persistent membrane bleb formed from the cortex of the embryo. DECON was defined as the loss of punctate mCherry-H2B signal within the decondensing chromatin. To minimize bleaching and maximize signal intensity of GFP-tagged spindle checkpoint components (NPP-21, PCH-2 and MAD-2), imaging was started just after NEBD as visualized by mCherry-H2B. Here, 8× 1μm steps were captured with 250ms GFP and 100ms mCherry exposures at 20s intervals.

Immunostaining was performed on worms 20–24 h after L4 stage, as described in (Russo et al., 2021). Gonad dissections were performed in 1× EBT (250 mM Hepes-Cl, pH 7.4, 1.18 M NaCl, 480 mM KCl, 20 mM EDTA, and 5 mM EGTA) + 0.1% Tween 20 and 20 mM sodium azide. An equal volume of 7.4% formaldehyde in EBT (final concentration was 3.7% formaldehyde) was added and allowed to incubate under a coverslip for 5 min. The sample was mounted on HistoBond slides (75 × 25 × 1 mm from Lamb), freeze-cracked, and incubated in methanol at −20°C for slightly more than 1 min and transferred to PBST (PBS with Tween 20). After several washes of PBST, the samples were incubated for 30 min in 1% bovine serum albumin diluted in PBST. A hand-cut paraffin square was used to cover the tissue with 50 μl of antibody solution. Incubation was conducted in a humid chamber overnight at 4°C. Slides were rinsed in PBST and then incubated for 2 h at room temperature with fluorophore-conjugated secondary antibody at a dilution of 1:500. Samples were rinsed several times and DAPI stained in PBST, then mounted in 13 μl of mounting media (20 M *N*-propyl gallate [Sigma-Aldrich] and 0.14 M Tris in glycerol) with a no. 1 1/2 (22 mm^2^) coverslip, and sealed with nail polish.

Mouse anti-FLAG [Sigma] primary antibodies were used for immunofluorescence at 1:1000 and Alexa-Fluor 488 anti-mouse (Invitrogen) secondary antibodies were used at 1:500. Three-dimensional image stacks were collected at 0.2-μm Z-spacing and processed by constrained, iterative deconvolution. Image scaling and analysis were performed using functions in the softWoRx software package. Projections were calculated by a maximum intensity algorithm. Composite images were assembled and some false coloring was performed with Adobe Photoshop.

### Quantification of NPP-21-GFP, GFP-MAD-2, and PCH-2-GFP-3XFLAG

Analysis was performed in Fiji. Quantification of fluorescence around mitotic chromosomes was quantified as described in (Defachelles et al., 2020). Sum intensity projections were generated and average fluorescence in the area around mitotic chromosomes was measured in Fiji. Background average fluorescence was measured in a 30-pixel band around this “cloud” and subtracted from the initial fluorescence intensity to determine the final value. In some of our movies to quantify PCH-2-GFP and localize NPP-21-GFP, identifying a clear metaphase plate was more difficult in AB than in P1 cells. Therefore, to ensure that we were performing these experiments at the same stage in mitosis in these two cell types, frames for the relevant analysis were normalized relative to NEBD and mitotic exit.

Quantification of unattached kinetochore signal was performed essentially as described for GFP-MAD-2 quantification in (Nelson et al., 2015). Maximum intensity projections of both mCherry-H2B and GFP fusion proteins were made after the pseudo-metaphase plate was generated. The image was rotated so the metaphase plate was vertical, channels were split, and the maximum GFP pixel was identified using the process function within a box on the unattached side of the metaphase plate. In the same x-plane, the maximum mCherry-H2B pixel was found. The width was changed to 12 pixels and the maximum GFP signal intensity was recorded in this 12 pixel window centered at the mCherry maxima. The background GFP signal was calculated by taking the average GFP intensity of a 4 pixel box in the same x-plane, 8 pixels away from the maximum mCherry on the opposite side of the pseudo-metaphase plate to the maximum GFP (i.e. the attached side). This background GFP was then subtracted from the maximum to measure the kinetochore bound GFP fusion intensity. This process was repeated at least 7x for each genetic background and treatment.

### Feeding RNA interference (RNAi)

RNA interference (RNAi) was performed by growing relevant worm strains on HT115 bacteria transformed with a vector allowing for IPTG inducible expression of dsRNA against the *zyg-1* gene. Bacterial strains containing this RNAi vector were cultured overnight at 37°C, centrifuged, and the pellet was re-suspended in 1/10 of the original volume. 50uL of concentrated culture was spotted onto a nematode growth medium (NGM) plate with 1mM IPTG and 50ug/uL of kanamycin and the RNAi spot was allowed to grow overnight at 37°C. Plates were stored at 4°C for up to 10 days.

L4 hermaphrodite worms were transferred to RNAi plates, allowed to incubate for 2–3 hours, and then transferred to fresh RNAi plates. Live microscopy was performed on embryos 22–26 hours after worms were picked to the *zyg-1* RNAi plate. HT115 bacteria transformed with pHSG298 (Clontech) was used as a control for *zyg-1*^*RNAi*^.

### Auxin treatment

Auxin treatment was performed on worms 20–24 hours after L4 stage by transferring worms to bacteria-seeded plates containing auxin or ethanol. The natural auxin indole-3-acetic acid (IAA) was purchased from Alfa Aesar (#A10556). A 100 mM stock solution in ethanol was prepared and was stored at 4°C for up to two months. 100ul of the auxin stock solution or ethanol was added to NGM plates (volume 10mls) to achieve a final concentration of 1mM.

To perform RNAi against *zyg-1* and auxin treatment, L4 hermaphrodite worms were transferred to RNAi plates, allowed to incubate for 2–3 hours, and then transferred to fresh RNAi plates for 20–22 hours. Worms were then transferred to RNAi plates containing either auxin or ethanol to incubate for 2–3 hours before performing live microscopy.

### Statistical Analysis

Data was analyzed using Prism for statistical significance. For Figures 2C, D and E, student’s t-test was used to assess significance. For all other data, one-way ANOVA with Šídák correction was used to assess significance.

## Supplementary Material

Supplement 1 **Supplemental Figure 1: Grayscale images of PCH-2-GFP in *AID::npp-21* strains without (top) and with TIR1 (bottom).** Area of enrichment indicated by yellow dotted circle. Scale bar indicates 5 microns.

Supplement 2 Supplemental Table 1: *C. elegans* strains used in this study

## Figures and Tables

**Table 1:** Viability of strains

Genotype and condition	Embryonic viability (number of embryos)	% Males
Control + ethanol	100% (2165)	0.05%
Control + auxin	100% (1799)	0.08%
*AID-3xFLAG::npp-21 Pgld-1::TIR-1-mRuby* + ethanol	96% (2245)****	2.3%****
*AID-3xFLAG::npp-21 Pgld-1::TIR-1-mRuby* + auxin	0.25% (1598)****	25%****
*pch-2::GFP-3xFLAG*	100% (3061)	0.06%
*AID-3xFLAG::npp-21 pch-2::GFP-3xFLAG*	100% (3373)	0.6%***
*AID-3xFLAG::npp-21 Psun-1::TIR-1-mRuby*	100% (2113)	1.7%****

A *** indicates a P value < 0.001 and a **** indicates a P value < 0.0001
