# Supplementary material for: NPP-21/TPR is required for developmental control of spindle checkpoint strength in C. elegans
Source: bioRxiv. 2026 Apr 24:2026.04.13.718277. Originally published 2026 Apr 15. Preprint. [Version 2] doi: 10.64898/2026.04.13.718277 (PMC13104992; doi:10.64898/2026.04.13.718277)
Supplement: Supplement 1 — Supplemental Figure 1: A. Images of AB and P1 cells expressing mCherry::H2B (magenta) and GFP::PH (green) at NEBD or OCC in control or zyg-1RNAi embryos. B. Grayscale images of mCherry::H2B in control or zyg-1RNAi embryos. Scale bars indicate 5 microns. [file media-1.pdf]

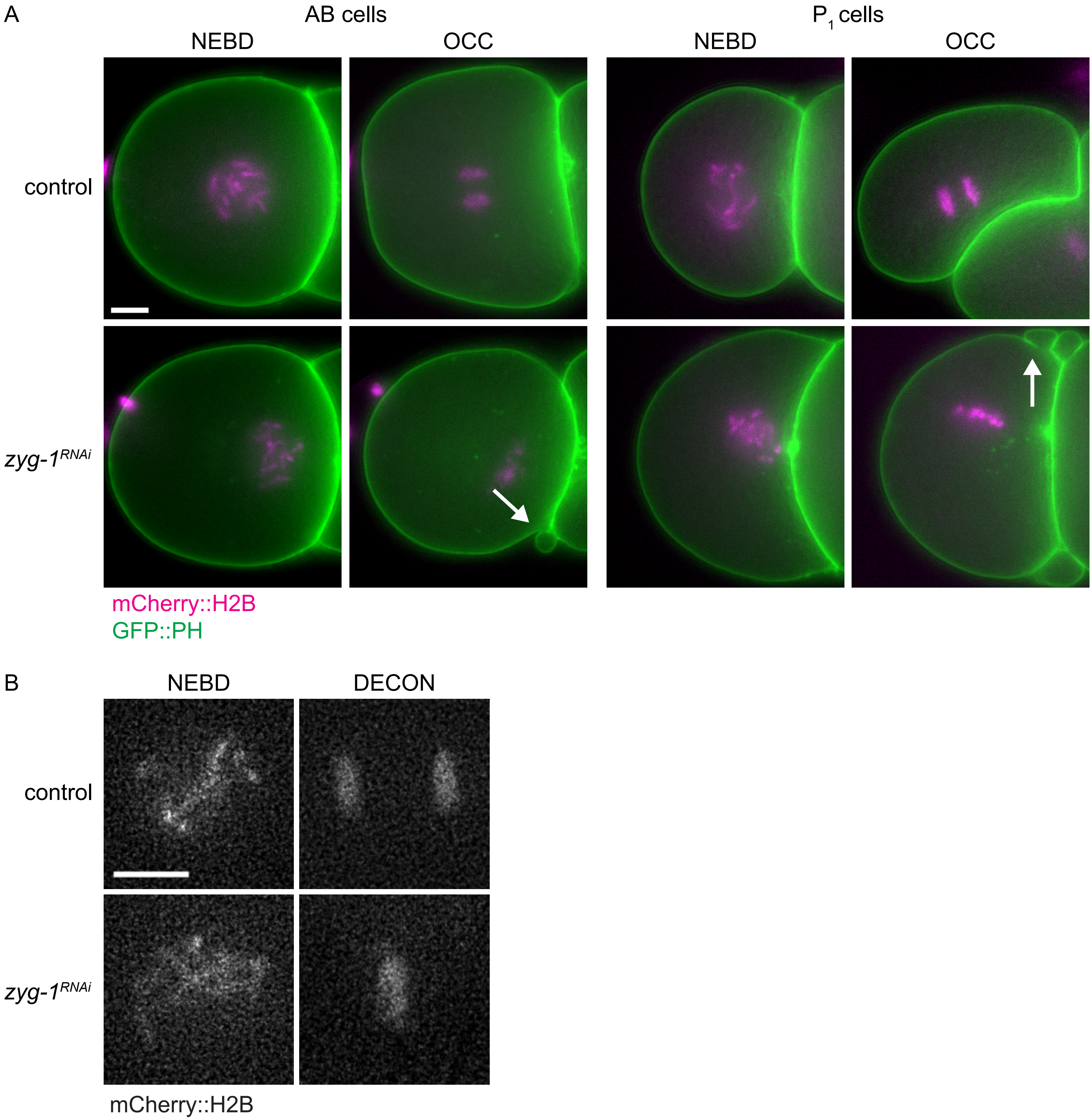

Supplemental Figure 1: Hallmarks of mitotic entry and exit in *C. elegans* embryos. A. Images of AB and P_1 cells expressing mCherry::H2B (magenta) and GFP::PH (green) at NEBD or OCC in control or *zyg-1^{RNAi}* embryos. B. Grayscale images of mCherry::H2B at NEBD or DECON in control or *zyg-1^{RNAi}* embryos. Scale bars indicate 5 microns.
