# Supplementary material for: NPP-21/TPR is required for developmental control of spindle checkpoint strength in C. elegans
Source: bioRxiv. 2026 Apr 24:2026.04.13.718277. Originally published 2026 Apr 15. Preprint. [Version 2] doi: 10.64898/2026.04.13.718277 (PMC13104992; doi:10.64898/2026.04.13.718277)
Supplement: Supplement 3 — Supplemental Table 1: C. elegans strains used in this study [file media-3.pdf]

Supplemental Table 1. ***C. elegans*** **strains used in this study**

**Strain Number**	**Genotype**
OD595/BHL539	*unc-119(ed3) III; ltIs37* [*pAA64; pie-1/mCherry::his-58; unc-119 (+)*] *IV; ltIs38* [*pAA1; pie-1/GFP::PH(PLC1delta1); unc-119(+)*]
BN1062/BHL114	*npp-21::GFP(bq1) bqSi189[pBN13(unc-119(+) Plmn-1::mCherry::his-58)] II; unc-119(ed3) III*
CA1503/BHL1119	*Pgld-1::TIR-1-mRuby(ieSi64) AID-3xFlag::npp-21(ie119) II*
BHL600	*unc-119(ed3) III; ltIs37* [*pAA64; pie-1/mCherry::his-58; unc-119 (+)*] *IV; ltIs52* [*pOD379; pie-1/GFP::MDF-2; unc-119 (+)*]
BHL664	*pch-2::GFP-3xFLAG(blt04, pCN94) II; unc-119(ed3) III; ltIs37* [*pAA64; pie-1/mCherry::his-58; unc-119 (+)*] *IV*
BHL1134	*Pgld-1::TIR-1-mRuby(ieSi64) AID-3xFlag::npp-21(ie119) II; unc-119(ed3) III; ltIs37* [*pAA64; pie-1/mCherry::his-58; unc-119 (+)*] *IV; ltIs38* [*pAA1; pie-1/GFP::PH(PLC1delta1); unc-119(+)*]
BHL1177	*Pgld-1::TIR-1-mRuby(ieSi64) AID-3xFlag::npp-21(ie119) II; unc-119(ed3) III; ltIs37* [*pAA64; pie-1/mCherry::his-58; unc-119 (+)*] *IV; ltIs52* [*pOD379; pie-1/GFP::MDF-2; unc-119 (+)*]
BHL1181	*AID-3xFlag::npp-21(ie119) pch-2::GFP-3xFLAG(blt04, pCN94) II; unc-119(ed3) III; ltIs37* [*pAA64; pie-1/mCherry::his-58; unc-119 (+)*] *IV*
BHL1195	*AID-3xFlag::npp-21(ie119) pch-2::GFP-3xFLAG(blt04, pCN94) II; unc-119(ed3) III; ltIs37* [*pAA64; pie-1/mCherry::his-58; unc-119 (+)*] *ieSi38 [Psun-1::TIR1::mRuby::sun-1 3'UTR, cb-unc-119(+)] IV*
