# Supplementary material for: NPP-21/TPR is required for developmental control of spindle checkpoint strength in C. elegans
Source: bioRxiv. 2026 Apr 15:2026.04.13.718277. Preprint. [Version 1] doi: 10.64898/2026.04.13.718277 (PMC13104992; doi:10.64898/2026.04.13.718277)
Supplement: Supplementary file 1 [file media-1.pdf]

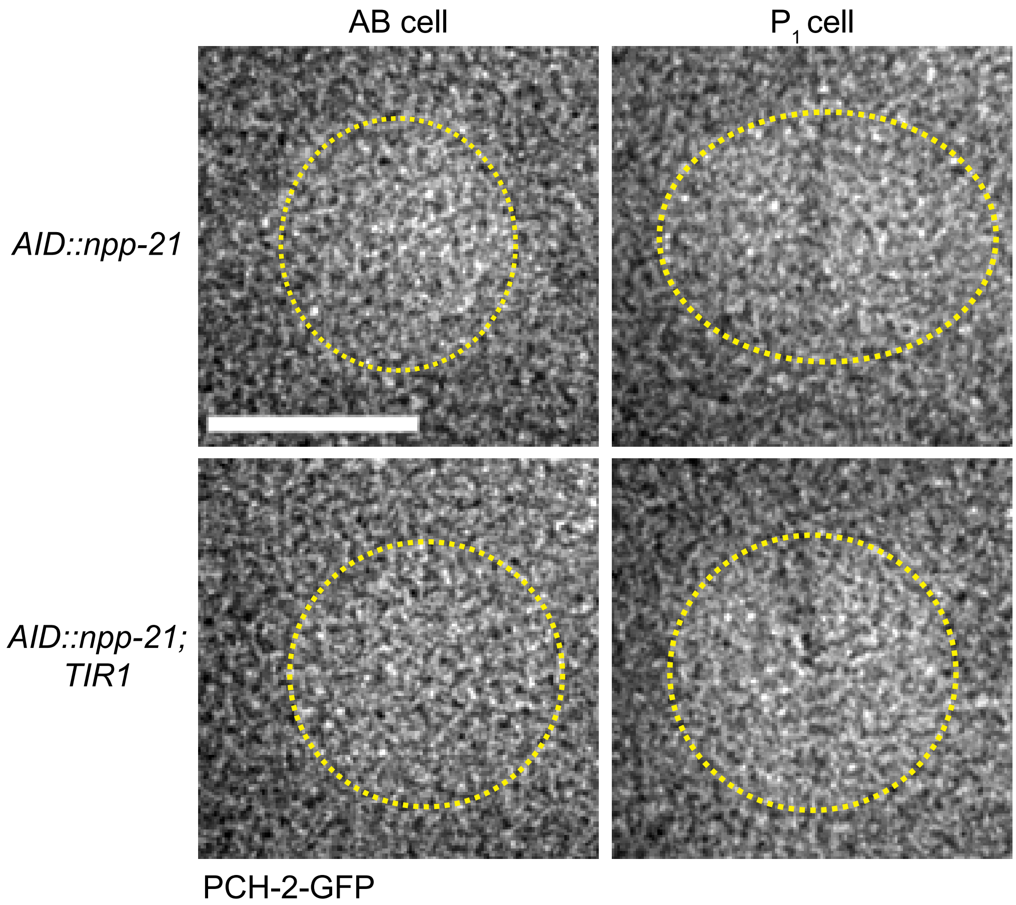

Supplemental Figure 1: Grayscale images of PCH-2-GFP in *AID::npp-21* strains without (top) and with TIR1 (bottom). Area of enrichment indicated by yellow dotted circle. Scale bar indicates 5 microns.
